# Hypoglycemic Effect of Hemp Seed Cake Protein Hydrolysates in Streptozotocin-Induced Diabetic Mice

**DOI:** 10.3390/foods15193432

**Published:** 2026-09-25

**Authors:** Ayten Solak, Daniela Miteva-Gyurova, Iliana Nacheva, Iliyan Manoylov, Svetla Dyankova, Penka Petrova, Petya Metodieva, Maria Doneva

**Affiliations:** 1 Institute of Cryobiology and Food Technologies, Agricultural Academy, 1407 Sofia, Bulgaria; daniela.miteva@ikht.bg (D.M.-G.); director@ikht.bg (I.N.); svetla.diankova@ikht.bg (S.D.); petya.metodieva@ikht.bg (P.M.); maria.doneva@ikht.bg (M.D.); 2 The Stephan Angeloff Institute of Microbiology (SAIM), Bulgarian Academy of Sciences, 1113 Sofia, Bulgaria; iliyanmanoylov@microbio.bas.bg (I.M.); ppetrova@microbio.bas.bg (P.P.)

**Keywords:** bioactive peptide, hemp seed cake hydrolysates, streptozotocin-induced diabetes, hypoglycemic effect

## Abstract

The potential of plant-based bioactive peptides to be used as an alternative or complementary therapy for the safe management of metabolic diseases such as diabetes is garnering increasing attention. In this in vivo study, the hypoglycemic effect of bromelain hydrolysates of hemp seed cake protein is analyzed in streptozotocin-induced diabetic mice. The results demonstrate that treatment of mice with the hydrolysate at a dose of 200 mg/kg/day for 4 weeks leads to a significant decrease in blood glucose levels (by 39%), serum triglyceride levels (16.44%), total cholesterol (13.50%), and low-density lipoprotein LDL (23.60%) compared to the untreated streptozotocin (STZ) group. The obtained values are comparable to those of the group treated with acarbose, which is used as a reference drug in the study. The applied hydrolysate significantly reduces serum alanine aminotransferase levels (by 39.38%) compared to the values of the untreated STZ group, while the change in superoxide dismutase levels was comparable to that of the group treated with acarbose. This study demonstrates the capacity of the applied hydrolysate to improve hyperglycemic conditions and reveals opportunities for future research into its potential as a functional ingredient.

## 1. Introduction

Diabetes mellitus (DM) is one of the most prevalent metabolic disorders globally, characterized by chronic hyperglycemia resulting from impairment in insulin secretion, insulin action, or both. Diabetes mellitus includes two main types: Type 1 DM, which results from insulin deficiency, and Type 2 DM, which is mainly due to insulin resistance. According to recent data [1], approximately 589 million people worldwide aged 20–79 are currently living with DM, of whom 66 million are in Europe, and 482,000 in Bulgaria. The number of patients globally is expected to rise to 853 million people by the year 2050 [1]. DM poses a profound public health challenge, not only due to its increasing prevalence, but also because of the association of the resulting chronic hyperglycemia with severe complications (such as cardiovascular diseases, neuropathy, retinopathy, and renal failure), which threaten human health and are considered causes of morbidity and mortality in diabetic patients [2]. In 2024, DM was responsible for an estimated 3.4 million deaths according to International Diabetes Federation data [1]. The increasing prevalence, severe complications, and chronic course make DM a major human health problem.

Despite advances in the treatment of DM that achieve effective control of blood sugar levels, many patients experience adverse side effects and limited long-term efficacy, which prompts scientists to search for alternative therapeutic strategies [3]. Consequently, recent decades have seen a growing interest in researching natural bioactive compounds with potential hypoglycemic properties that could be used in alternative or complementary therapies for the safe management of metabolic disease and the prevention of complications [4,5]. Among these, peptides obtained through the hydrolysis of plant proteins are some of the most frequently reported natural compounds with potential for modulating glucose metabolism and improving insulin sensitivity [6,7,8,9]. In addition to their hypoglycemic properties via mechanisms such as enzyme inhibition, improved insulin signaling, and modulation of glucose absorption, bioactive peptides possess other health benefits; there are reports of antihypertensive, antioxidant, anticoagulant, hepatoprotective, anti-inflammatory, antimicrobial, and other effects [10,11,12,13].

Hemp seeds (*Cannabis sativa* L.) are primarily processed for hemp oil extraction, leaving hemp seed cake as a major by-product. This cake contains a significant amount of protein (ranging from 30% to 50% dry weight, depending on the cultivar used and the method of oil extraction) and serves as a valuable resource for producing bioactive peptides obtained through hydrolysis [14]. Hemp protein hydrolysates (HPHs) have attracted substantial attention in recent years due to their significant biological activity, including a hypoglycemic effect [15,16]. The hypoglycemic effect of HPHs may be attributed to their established inhibitory activity against dipeptidyl peptidase IV (DPP-IV) (responsible for modulating incretin levels), α-glucosidase, and α-amylase (critical for carbohydrate catabolism), alongside their ability to modulate insulin signaling pathways and glucose transport. Specific hemp hydrolysates prepared with alcalase, pepsin, trypsin, protamex, neutrase, flavourzyme, papain, bromelain, etc. are reported in the literature. These enzymes hydrolyze peptide bonds and release peptides of various sizes and properties, with the physiological function of the active peptide believed to be closely linked to its parent protein matrix, amino acid profile, and the protease used for hydrolysis [7,15,17].

It has been established that hydrolysis of hemp seed protein with the enzyme alcalase releases bioactive peptides with a high content of hydrophobic amino acids that suppress α-glucosidase activity [18]. In another study, peptide fractions (rich in arginine) were obtained via tryptic hydrolysis, which can modulate glucose and lipid metabolism by inhibiting α-amylase and lipase and by regulating the AMPK-AKT1/PPARG/HMGCR metabolic pathway [19]. Controlled enzymatic hydrolysis using pancreatin, trypsin, papain, bromelain, thermolysin, compound protease, alkaline protease 37071, corolase 7089, corolase 8000, and amano AY50C has yielded hydrolysates that inhibit α-glucosidase and transmembrane glucose transport in Caco-2 cells [20]. Furthermore, hydrolysis with pepsin [21] or with alcalase and flavourzyme, both with and without subsequent digestion according to the INFOGEST protocol [22], has produced peptides with enhanced inhibitory activity against DPP-IV. In addition to a hypoglycemic effect, hemp hydrolysates exhibit anti-inflammatory, antihypertensive, hypolipidemic, and neuroprotective properties [16,17]. High antioxidant activity has been reported (scavenging of DPPH and ABTS radicals, increasing the reducing capacity, Fe^2+^ chelation, etc.) [15], and according to [23], antioxidant supplements may be useful in reducing the risk of diabetic complications.

Data on the biological activity of HPHs have largely been obtained from *in silico* and *in vitro* analyses [9,15]. Because these assays do not fully replicate the physiological complexity of a living system, in vivo studies are essential to validate their therapeutic potential and determine their efficacy in the prevention and management of hyperglycemia. Experimental models of STZ-induced diabetes in rodents are thought to mimic human hyperglycemia characteristic of Type 1 DM or, when using specific protocols, Type 2 DM. Models with a high single dose exhibit almost complete destruction of β-cells, leading to insulin deficiency and hyperglycemia, serving as an analog for Type 1 DM. Low multiple-dose models induce partial β-cell destruction and a milder diabetic state, whereas a high-fat diet (prior to STZ administration) can be used to model Type 2 DM, where insulin resistance is combined with impaired insulin secretion [24]. The influence of various active compounds, particularly hemp hydrolysates, on the induced changes in the model can help determine their potential as antidiabetic agents. Moreover, this model has been widely adopted for the evaluation of hypoglycemic agents [25].

The present study aims to evaluate the hypoglycemic effect of bromelain hydrolysates of hemp seed cake protein in mice with STZ-induced diabetes, providing data on their potential application as a functional food ingredient for the control of hyperglycemia.

## 2. Materials and Methods

### 2.1. Chemicals

Cold-pressed hemp seed (*Cannabis sativa* L., ssp. *sativa*) oil cake (with particle sizes ˂ 0.500 mm, protein content −29.72%) was obtained from ViM Production Ltd., Stryklevo village, Ivanovo Municipality, Ruse Province, Bulgaria. The hemp seeds originated in Germany and comply with the strict delta-9-tetrahydrocannabinol (THC) limits under Commission Regulation (EU) 2023/915 [26]. Bromelain (EC 3.4.22.32; 256.475 U/g) was sourced from SERVA Electrophoresis GmbH, Heidelberg, Germany. Streptozotocin (STZ, ALX-380-010-M100) was purchased from Enzo Life Sciences, Farmingdale, NY, USA. Acarbose was purchased from Merck KGaA (Darmstadt, Germany). The cholesterol assay kit (ab65390), HDL and LDL/VLDL assay kit, alanine aminotransferase assay kit (ab282882-Mouse ALT SimpleStep ELISA^®^ Kit), superoxide dismutase (SOD) activity assay kit (ab65354), and triglyceride assay kit (ab65336) were purchased from Abcam (Cambridge, UK). Accu-Chek Performa test strips were from Roche Diagnostics GmbH (Mannheim, Germany). All other reagents (analytical grade) were supplied by Merck KGaA (Darmstadt, Germany).

### 2.2. Hemp Protein Hydrolysates

Protein hydrolysates were obtained from hemp cake according to the method described by [27], with slight modifications. Briefly, hemp seed samples were suspended in 0.1 M phosphate buffer (pH 6.5) to obtain a mixture with a protein concentration of 6.25 g/100 mL. Bromelain was added to the mixture at a concentration of 1.3% relative to the protein content. Hydrolysis was carried out at 39 °C for 120 min in a water bath. Following lyophilization (LYOBETA 6PL, Teslar, Barcelona, Spain), the obtained hydrolysates were stored at −28 °C until further uses. The hydrolysates (degree of hydrolysis—30.71 ± 1.24%, as determined by the method described in [28]) had a total protein content 72.94 ± 0.18% (Kjeldahl, N × 6.25) and a moisture content of 3.21 ± 0.34% (Moisture Analyzer DBS 60-3, Kern & Sohn GmbH, Balingen-Frommern, Germany), with an established *in vitro* inhibitory activity against α-amylase of 60.72 ± 1.68%, as determined by the method described in [29] and against α-glucosidase of 82.81 ± 0.23%, as determined by the method described in [30] at a concentration of 2.50%.

### 2.3. Animal Experiments

All animals included in the present study were handled in accordance with the Bulgarian national regulations and the Guidelines for the Care and Use of Laboratory Animals of the European Union [31]. The manipulations were approved by the animal care commission at the Bulgarian Food Safety Agency, approval protocol Nr. 438/15.07.2025. The mice were bred and kept at the animal facility of the Institute of Microbiology, Bulgarian Academy of Sciences and housed at 20–22 °C with a 14:10 h light/dark cycle.

### 2.4. Subacute Toxicity Study

To evaluate the subacute toxicity of hemp hydrolysates, 30 male C57Bl/6J mice (20–24 g) aged 8–10 weeks were used. The animals were divided into five groups of six mice each (*n* = 6/cage). Group 1, designated as healthy, served as the control and was treated p.o. (orally) with 10 mL/kg BW tap water. Group 2, the positive control group, was treated with 100 mg/kg acarbose (reference drug, α-glucosidase inhibitor). Groups 3, 4, and 5 were treated with 100, 200, and 400 mg/kg body weight of hemp hydrolysates, respectively, dissolved in 200 μL tap water. Survival, changes in behavior, toxicological symptoms (piloerection, salivation, respiratory disorders, diarrhea, tremor, changes in motor activity), and the body weights of the animals were monitored over a 29-day period. After euthanasia by cervical dislocation, the relative weights of the spleen and liver were determined. The relative weights were calculated using the following formulas:

(1) $$Relative\;Spleen\;Weight\;\left(\%\right)=\;\frac{spleen\;weight}{total\;weight}\times 100$$

(2) $$Relative\;Liver\;Weight\;\left(\%\right)\;=\;\frac{liver\;weight}{total\;weight}\times 100$$

### 2.5. Groups and Experimental Design

A total of 36 male C57Bl/6J mice (20–24 g), aged 8–10 weeks, were used in the experiments. Food and water were provided to the animals ad libitum. The mice were randomly separated into six groups (six animals per group, *n* = 6/cage), as follows:

- Group 1—STZ—streptozotocin-challenged group.
- Group 2—Healthy—untreated group.
- Group 3—STZ group treated with 100 mg/kg acarbose.
- Group 4—STZ group treated with 100 mg/kg of hemp hydrolysates.
- Group 5—STZ group treated with 200 mg/kg of hemp hydrolysates.
- Group 6—STZ group treated with 400 mg/kg of hemp hydrolysates.

In order to follow up on the blood glucose levels and the body weight changes of the mice, a Data Gathering Protocol (DGP) was developed.

At 7 o’clock in the morning on days 1, 8, 15, 22, 29, and 36 of the experiment, the food was removed from the cages (water was available ad libitum). After 4 h of fasting (between 11:00 and 11:30 o’clock), the DGP was performed. The mice were anaesthetized with 2% isoflurane (100 mL/min flow), and 5 mg/kg of meloxicam was administered via s.c. injection. Next, the animals were weighed, and 250 μL of blood was taken from the retro-orbital sinus. Blood glucose levels were measured using an Accu-Chek Performa blood glucose monitoring system and Accu-Chek Performa test strips. Lastly, an i.p. injection of 250 μL of warm saline was administered to the animals, to compensate for the blood loss.

Groups 1, 3, 4, 5, and 6 were challenged with 40 mg/kg streptozotocin (STZ, ALX-380-010-M100, Enzo Life Sciences, Farmingdale, NY, USA) i.p., dissolved in 0.1 M sodium citrate buffer, pH 4, for diabetes induction from day 1 to day 5 (5 consecutive days). From day 1 to day 5, Group 2 received a saline injection. The injections on day 1 were administered post-GDP, whereas those from day 2 to day 5 were performed after a 4 h fasting period. Mice with a blood glucose level above 14 mmol/L on day 8 were considered diabetic and were included in the study.

From day 8 to day 36 (28 days), Group 1 and Group 2 received 10 mL/kg BW (200 μL) of tap water per os (p.o.). During this period, Groups 3, 4, 5, and 6 received the corresponding treatments dissolved in 200 μL of tap water per os (p.o.). Mice from all groups were subjected to the DGP on days 1, 8, 15, 22, 29, and 36.

The experimental design is shown in Figure 1.

On the final day of the experiment, after the DGP was performed, all animals were euthanized by cervical dislocation, and their spleens and liver were weighed for toxicological analysis, based on their relative weights (represented as percentages). The relative weights were calculated using Formulas (1) and (2).

### 2.6. Serum Analyses

All blood samples were collected from the retro-orbital sinus of the experimental animals and left for 2 h at 4 °C for clot formation. The clotted blood was centrifuged twice at 1000× *g*, and the obtained sera were kept at −80 °C for assays with commercial kits, which include analysis of the levels of cholesterol (ab65390-Cholesterol Assay Kit—HDL and LDL/VLDL), alanine aminotransferase (ab282882-Mouse ALT SimpleStep ELISA^®^ Kit, Abcam, Cambridge, UK), SOD activity (ab65354-Superoxide Dismutase Activity Assay Kit, Abcam, Cambridge, UK (Colorimetric), and triglycerides (ab65336-Triglyceride Assay Kit, Abcam, Cambridge, UK). All kits were used according to the manufacturers’ instructions.

### 2.7. Statistical Analysis

All data are expressed as mean ± standard error of the mean (SEM). One-way ANOVA followed by Dunnett’s multiple comparisons test was performed using GraphPad Prism version 8.0.0 for Windows (GraphPad Software, San Diego, CA, USA, www.graphpad.com., accessed on 10 November 2025). A *p*-value of less than 0.05 (*p* < 0.05) was considered statistically significant.

## 3. Results and Discussion

### 3.1. Subacute Toxicity Study

In the subacute toxicity study, a 100% survival rate was recorded for the animals (Figure 2).

Following oral administration of the three experimental doses of hemp hydrolysates (100, 200, and 400 mg/kg), no clinical signs of intoxication or changes in behavior were observed. There were no changes in body weight or the relative weights of the spleen and liver in the groups treated with hemp hydrolysates compared to the control groups––both healthy and treated with acarbose (reference drug, α-glucosidase inhibitor)––Figure 3.

Changes in organ weight are generally considered to be a sensitive indicator of chemically induced changes in organs, and comparing organ weights between treated and untreated animals is traditionally used to evaluate the toxic effect of the tested substance [32]. In the conducted study, no significant difference was observed in the relative weights of the spleen and liver among the groups treated with the three experimental doses of hemp hydrolysates—100, 200, and 400 mg/kg. Alongside this, it should be mentioned that, in the group treated with the hemp hydrolysate at a dose of 400 mg/kg, the relative weight of the liver reached the highest value, although, as already mentioned, the difference compared with the other groups was not significant (*p* > 0.05).

### 3.2. Effects of Hemp Seed Cake Hydrolysates on Body Weight and Relative Spleen and Liver Weights in Mice

Figure 4 presents the data for the percentage delta (∆) change in body weight among the different experimental groups. The healthy control animals demonstrate the expected body weight gain (by ~ 5%) and show a positive value. In contrast, a drastic decrease in body weight (~−19%) is observed in the STZ group, with the difference between the two groups being statistically significant (*p* < 0.001). Treatment with acarbose shows a trend towards weight stabilization, with a significant difference compared to the STZ group (*p* < 0.05). When analyzing the effect of the studied hydrolysates on body weight, no positive result was observed upon administration of the 100 mg/kg and 400 mg/kg doses. At these doses, the hemp hydrolysates failed to compensate for the weight loss, with their values remaining close to those of the STZ group. Among the experimental groups, only treatment with 200 mg/kg of hemp hydrolysate (STZ + 200 mg/kg group) exerted a stabilizing effect, and the results obtained are comparable to those of the group treated with acarbose.

In the groups with streptozotocin-induced diabetes, the experimental animals show a profound loss of body weight [33,34]. Streptozotocin—an analog of N-acetylglucosamine—is transported into pancreatic β-cells via GLUT-2 and causes β-cell toxicity, leading to insulin deficiency. Insulin deficiency, in turn, triggers the depletion of protein and lipid reserves, which leads to weight loss in animal models [35,36]. After 28 days of treatment with hemp hydrolysates at the three experimental doses, only the STZ + 200 mg/kg group showed a deceleration of body weight loss, with the results (Δ) being comparable to the acarbose-treated group. No positive result was observed with the administration of the 100 mg/kg and 400 mg/kg doses. They failed to compensate for the weight loss, with the values in these groups remaining close to the result for the STZ group.

Analysis of relative spleen weights revealed a significant increase (*p* < 0.0001) in the value for the untreated STZ group compared to the healthy group. An increase in the relative weight of the spleen is also observed in the group treated with hemp hydrolysate at a dose of 100 mg/kg, and the result does not differ from the STZ group. In the groups treated with acarbose and hemp hydrolysates at doses of 200 and 400 mg/kg (groups STZ + acarbose, STZ + 200 mg/kg, and STZ + 400 mg/kg), a significant difference (*p* < 0.001, *p* < 0.001, and *p* < 0.01, respectively) compared to the STZ group and an insignificant difference compared to the healthy control group is observed.

Treatment with hemp hydrolysates at doses of 200 and 400 mg/kg significantly alleviates (*p* < 0.001 and *p* < 0.01, respectively) the increased relative spleen weight observed in the untreated STZ group. The results obtained are comparable to the reference group treated with acarbose. The 100 mg/kg dose of the hemp hydrolysate does not lead to improvement in the condition, with the result being comparable to the STZ group, showing no significant difference (*p* > 0.05). There are no reports in the literature of enlarged spleens observed in STZ-induced diabetic animals. Conversely, atrophic changes in the organ and a decrease in its relative weight are reported [37,38]. In our experiments, the serious loss of body weight is the reason for the higher relative weight values of the organ. The occurrence of oxidative stress and the activation of inflammatory pathways are other probable causes for the observed high relative spleen weight values. It should be mentioned that splenomegaly is a condition encountered in some chronic diseases, including type 2 diabetes in people with metabolic syndrome [39]. Spleen enlargement and dysfunction in diabetes are also reported in a study [40] in db/db experimental mice.

The relative liver weight shows a substantial increase (*p* < 0.0001) in the STZ group compared to the healthy control group. Upon treatment with hydrolysates at doses of 100 mg/kg and 400 mg/kg, the relative liver weight remains high compared to the healthy control group and the group treated with acarbose, showing no significant differences from the untreated STZ group. In the group treated with hemp hydrolysate at a dose of 200 mg/kg, the relative liver weight is comparable to the values obtained for the acarbose group and is significantly lower than that of the STZ group (*p* < 0.01).

These observations align with previous literature indicating that STZ-induced diabetes is associated with a marked increase in absolute and relative liver weights, primarily due to increased oxidative stress and an aberrant inflammatory response resulting from β-cell and direct systemic toxicity of streptozotocin [41]. Hepatic processes during hyperglycemia are associated with glycation, fibrotic changes, or hypertrophic changes [25,42,43] and lead damage its architecture. In the current study, treatment with 200 mg/kg hemp hydrolysates successfully attenuated these increases in relative liver weight, with results comparable to those of acarbose.

### 3.3. Effects of Hemp Seed Cake Hydrolysates on Blood Glucose Levels in Experimental Mice

Animals treated with streptozotocin at the doses and number of exposures specified in the experimental design exhibit sustained hyperglycemia; therefore, the model used is suitable for investigating hemp hydrolysates for hypoglycemic effects. Hyperglycemia manifests after the fifth day from the first streptozotocin exposure, with all STZ groups maintaining levels ≥ 14 mmol/L after the eighth day. In the untreated STZ group, there is a progressive increase in blood glucose levels, reaching 30.21 ± 4.37 mmol/L on day 36 (Figure 5). The results of the analysis of the hypoglycemic effect of hemp hydrolysates show that, at the three tested doses of hemp hydrolysates (100, 200, and 400 mg/kg), there is an effective and dose-dependent significant reduction (up to 27.27 ± 4.39 mmol/L, 18.48 ± 5.87 mmol/L, and 15.23 ± 4.87 mmol/L on day 36, respectively) in glucose levels compared to the untreated STZ group. Glucose levels at doses of 200 mg/kg and 400 mg/kg are comparable to those of the acarbose-treated group and are about two times lower than those of the STZ group, but remain higher than those of the healthy control group (5.42 ± 1.39 mmol/L and 5.15 ± 0.52 mmol/L on days 8 and 36, respectively).

The use of plant-derived bioactive peptides as an alternative or complementary therapy for the management of DM is gaining increasing popularity [6]. In the present study, the hydrolysates we used were obtained by enzymatic hydrolysis with bromelain (with a degree of hydrolysis 30.71 ± 1.24%) and exhibit *in vitro* inhibitory activity against α-amylase (IC_50_ = 3.98 mg/mL) and α-glucosidase (IC_50_ = 3.43 mg/mL). The results from the analysis show that our hydrolysates, administered at doses of 200 mg/kg and 400 mg/kg to diabetic mice, exhibit a hypoglycemic effect comparable to that of acarbose. It has been demonstrated that acarbose used in STZ-induced diabetes in mice at a dose of 50 mg/kg for 14 days reduces blood glucose levels by approximately 150% [44].

Bromelain is a cysteine peptidase with endopeptidase action that exhibits specificity toward certain amino acid residues such as Gly, Arg, and Lys [45]. In an extensive review, Tacias-Pascacio et al. [4] discuss peptides from various sources obtained through hydrolysis with bromelain (alone or in combination with other proteases) and point out that, regardless of the protein source used, bromelain generates peptides (rich in hydrophobic amino acids and arginine-rich sequences) with excellent antidiabetic activity. On the other hand, the literature contains a number of *in vitro* and *in silico* analyses regarding the inhibitory activity against α-glucosidase, α-amylase, and DPP-IV, which indicate the potential for a hypoglycemic effect of hemp protein hydrolysates obtained using other enzymes [15,17,46]. It is believed that peptide sequences containing Trp, Arg, and Tyr at the C-terminus have a high probability of binding to the active site of α-amylase; Tyr, Phe, Lys, and Arg play a key role in binding to the active site of α-glucosidase; and Leu, Val, His, Phe, Trp, and Tyr are essential for the inhibition of DPP-IV [45]. Specific peptide sequences in hemp seed hydrolysates such as LR, PLMLP, FLLWETYR, FFFYLLDR, DNNYAWWR, FY, FR, TGLGR, and SPVI exhibit inhibitory activity against α-glucosidase, α-amylase, or DPP-IV [15,47].

Our findings regarding the hypoglycemic effect of hydrolysates of hemp seed cake protein are in agreement with a previous in vivo study involving an alloxan-induced hyperglycemia model and non-dehulled hemp seed meal hydrolysates (with alkaline protease) [47]. In two randomized crossover trials, Mollard et al. [48] demonstrated the ability of hemp protein, similar to soy protein, to lower postprandial glucose and insulin concentrations.

### 3.4. Effects of Hemp Seed Cake Hydrolysates on Superoxide Dismutase (SOD) Activity in Experimental Mice

Figure 6 shows the results of SOD activity in the experimental animals. On the 8th day after the first exposure to STZ, the SOD activity in serum from the untreated STZ group is 53.77 ± 3.00% and reaches 64.17 ± 1.86% on the 36th day, and the difference compared with the healthy control group is statistically significant (*p* < 0.05). In the group treated with acarbose, the values (53.63 ± 4.94% on the 36th day) were significantly lower than the STZ group. The adaptive response to the strong systemic oxidative stress during acarbose treatment reduces the need for a compensatory increase in SOD activity, and it is comparable in value to the healthy group (55.37 ± 0.91% on day 8 and 57.6 ± 1.88% on day 36). Hemp hydrolysates at a dose of 200 mg/kg showed SOD activity at levels close to those of the healthy group, and the values are comparable to those of the acarbose-treated group.

Metabolic dysfunction in DM disrupts the balance between the generation of reactive oxygen species and the body’s antioxidant defense, leading to oxidative stress. The overproduction of free radicals and reactive oxygen species can cause destructive cellular alterations through the oxidation of critical biopolymers such as DNA, membrane lipids, structural proteins, and enzymes [2,49]. Patients with diabetes exhibit high levels of oxidative stress markers and reduced antioxidant defense [2]. In this study, we observe an unexpected, yet statistically significant (*p* < 0.05) increase in the activity of the antioxidant enzyme SOD in the STZ group compared to the healthy group. Our results differ from the study by [50], which reports a decrease in SOD levels. The reasons for this may include interspecies differences and the applied dose of STZ used to induce diabetes. Our results can be explained by a compensatory response of the organism. Such an explanation is shared by researchers who have observed high SOD levels in diabetic patients (without complications) compared to healthy controls [51,52]. In this study, the hydrolysates we used did not change the SOD levels compared to the STZ group. However, the literature contains data on the high antioxidant activity of hemp protein hydrolysates in *in vitro* assays, as well as their ability to lower ROS production and increase antioxidant enzymes in cell cultures and in hypertensive rats [15,49,53].

### 3.5. Effects of Hemp Seed Cake Hydrolysates on Alanine Aminotransferase (ALT) Activity in Experimental Mice

Figure 7 shows the results for the effects of hemp seed cake hydrolysates on alanine aminotransferase (ALT) activity in experimental mice. As can be seen from the figure, all groups with STZ-induced diabetes on day 8 have higher values compared to the healthy control group. On day 36, ALT values were highest in the untreated STZ group (662.59 ± 65.35 pg/mL), with a significant difference from the healthy control (*p* < 0.0001). When treated with 200 mg/kg, ALT levels (401.61 ± 44.35 pg/mL) were comparable to those of the acarbose group (421.38 ± 63.71 pg/mL) and significantly lower than those of the STZ group (*p* < 0.0001).

The ALT enzyme is a marker of liver function. Elevated ALT levels are indicative of liver damage associated with hepatocyte destruction. In animals with STZ-induced diabetes, there is a significant increase in serum ALT [36,50], which is a key marker for diabetic hepatopathy (associated with direct hepatotoxicity, oxidative stress, inflammation, cell destruction, and increased gluconeogenesis). The decrease in serum ALT levels (by 39.38%) in the group treated with hemp hydrolysates (STZ + 200 mg/kg) compared to the STZ group reveals a positive effect of hemp hydrolysate on the liver.

### 3.6. Effects of Hemp Seed Cake Hydrolysates on Triglycerides and Cholesterol in Experimental Mice

Elevated cholesterol and triglyceride levels also contribute to the development and progression of microvascular and macrovascular complications in DM. Figure 8 presents the results for triglyceride levels. In the untreated STZ group, a progressive increase in triglyceride levels was observed, from 4.7 ± 0.83 mM (on day 8) to 9 ± 0.94 mM (on day 36), which indicates a deepening of the metabolic imbalance. Compared to the healthy control, the levels are nearly two times higher (*p* < 0.0001). Treatment with acarbose significantly limits (*p* < 0.001) the progressive increase in triglyceride levels observed with STZ. At day 36, levels are significantly lower than those of the untreated STZ group, stabilizing around 6.5 ± 0.56 mM. When treated with hemp hydrolysate at a dose of 200 mg/kg, a decrease in triglyceride levels is observed (by 16.44%), which is a significant hypolipidemic effect compared to the STZ group. The obtained values (7.52 ± 0.98 mM) show a significant difference compared to the healthy control (4.37 ± 0.45 mM) but are comparable to the group treated with acarbose.

Figure 9 shows the results for LDL/VLDL, total cholesterol, and HDL. As can be seen from the results, LDL/VLDL levels in the STZ group (0.89 ± 0.05 μg/μL) are almost twice as high (*p* < 0.0001) as in the healthy control group (0.48 ± 0.03 μg/μL). In the acarbose-treated group, there is a significant decrease (by 13.33%) in LDL/VLDL levels compared to the STZ group. The hemp hydrolysate-treated group showed low values (0.68 ± 0.03 μg/μL) compared to the acarbose group and the STZ group, with the latter difference being significant (*p* < 0.0001). Total cholesterol values in the STZ group reached 1.63 ± 0.06 μg/μL on day 36 and are significantly higher (*p* < 0.0001) than the healthy control group (0.92 ± 0.09 μg/μL). The hemp hydrolysate-treated group (at a dose of 200 mg/kg) had significantly lower values (1.41 ± 0.06 μg/μL) compared to the STZ group, and the result is comparable to the acarbose-treated group. Higher HDL values (0.73 ± 0.01 μg/μL) are observed in the STZ group compared to the healthy control group (0.44 ± 0.07 μg/μL). Treatment with acarbose and hemp hydrolysates maintained high HDL values compared to the healthy control group and shows no significant difference compared with the STZ group (*p* > 0.05).

DM is known to be associated with a large number of lipid abnormalities. Hyperlipidemia occurs with chronic progression in animal models of STZ-induced diabetes. Increased levels of LDL/VLDL and total cholesterol and decreased levels of HDL are observed [36]. In our study, the use of hemp hydrolysate effectively reduces triglyceride, LDL/VLDL, and total cholesterol levels (by 16.44%, 23.60%, and 13.50%, respectively). Our results confirm the positive effect of hemp protein hydrolysates on dyslipidemia reported in previous studies by other authors in a high-fat diet-induced obese mice model [19]. The high HDL values obtained in the STZ group contradict a study in animals with STZ-induced diabetes in which HDL levels are low, which the authors attribute to impaired HDL biogenesis [54]. A publication by [55] reported cases of high levels of total HDL in men with type 1 DM compared to healthy control subjects.

## 4. Conclusions

The results from this study show that bromelain hemp cake protein hydrolysate, used at a dose of 200 mg/kg for the treatment of C57Bl/6J mice with STZ-induced diabetes, has hypoglycemic and hypolipidemic effects. The observed significant reduction in blood glucose levels, serum triglyceride levels, total cholesterol, and LDL in the STZ + 200 mg/kg hydrolysate group compared to the untreated STZ group is comparable to the results obtained when treating the groups with acarbose (used as a reference drug, α-glucosidase inhibitor). When treated with the hydrolysate, a significant decrease in the level of serum ALT is observed compared to the values of the untreated control STZ group, as well as normalization of the relative liver weight. When treated with hemp hydrolysate, SOD levels are comparable to those of the group treated with acarbose.

This study demonstrates the capacity of the applied hemp cake hydrolysate to improve conditions of hyperglycemia and dyslipidemia in a mouse model of induced diabetes. Further research regarding the chemical characterization and evaluation of active peptides, followed by studies on its digestibility, stability, safety, and efficacy, will complete the picture regarding the potential of the hydrolysate and of the possibility of using it as a functional ingredient.

## Figures and Tables

**Figure 1:**
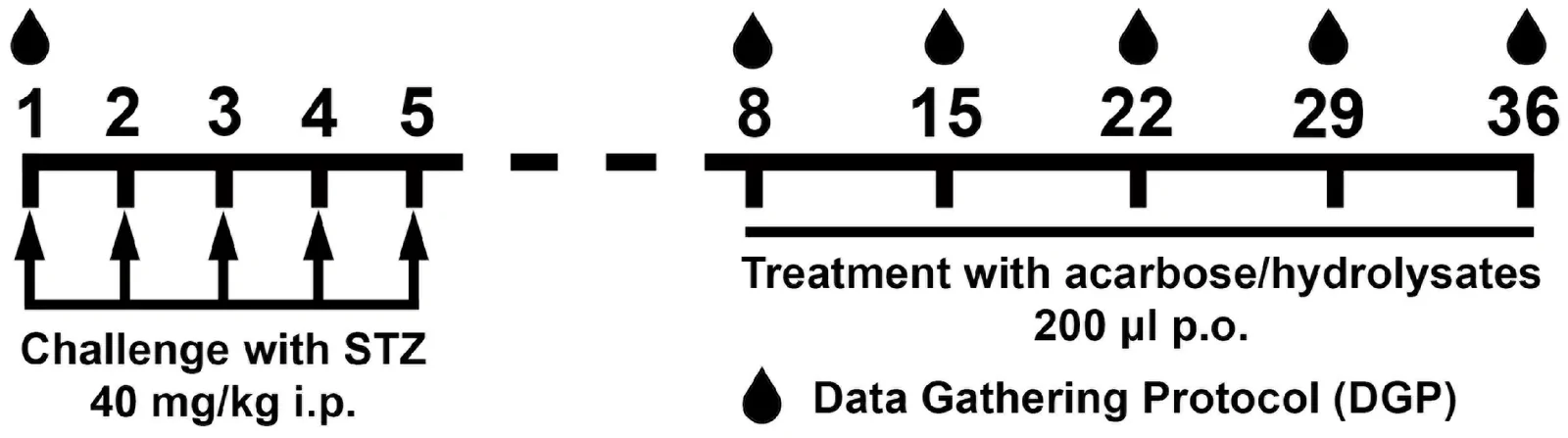
Experimental design.

**Figure 2:**
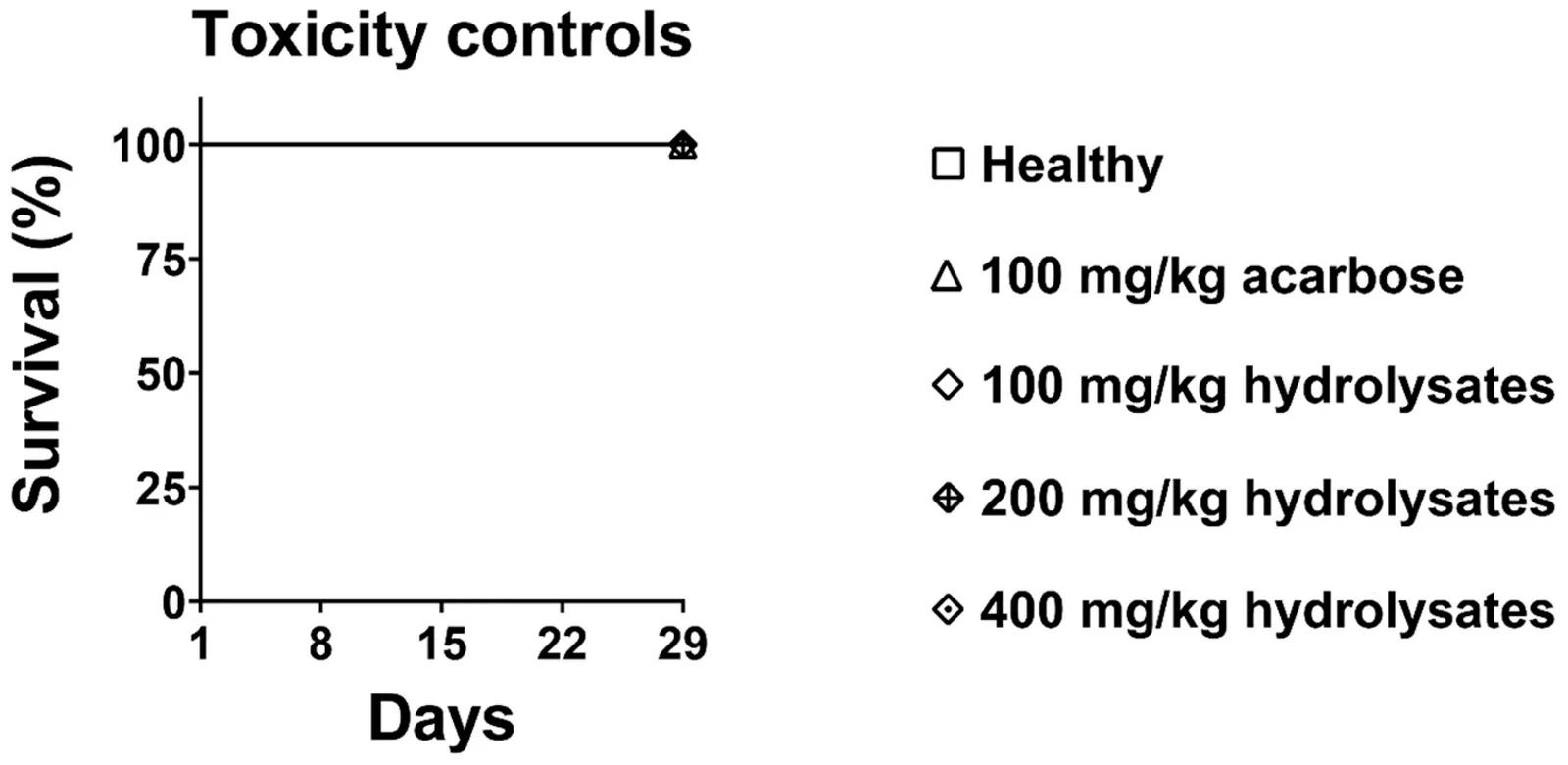
Animal survival in the subacute toxicity study.

**Figure 3:**
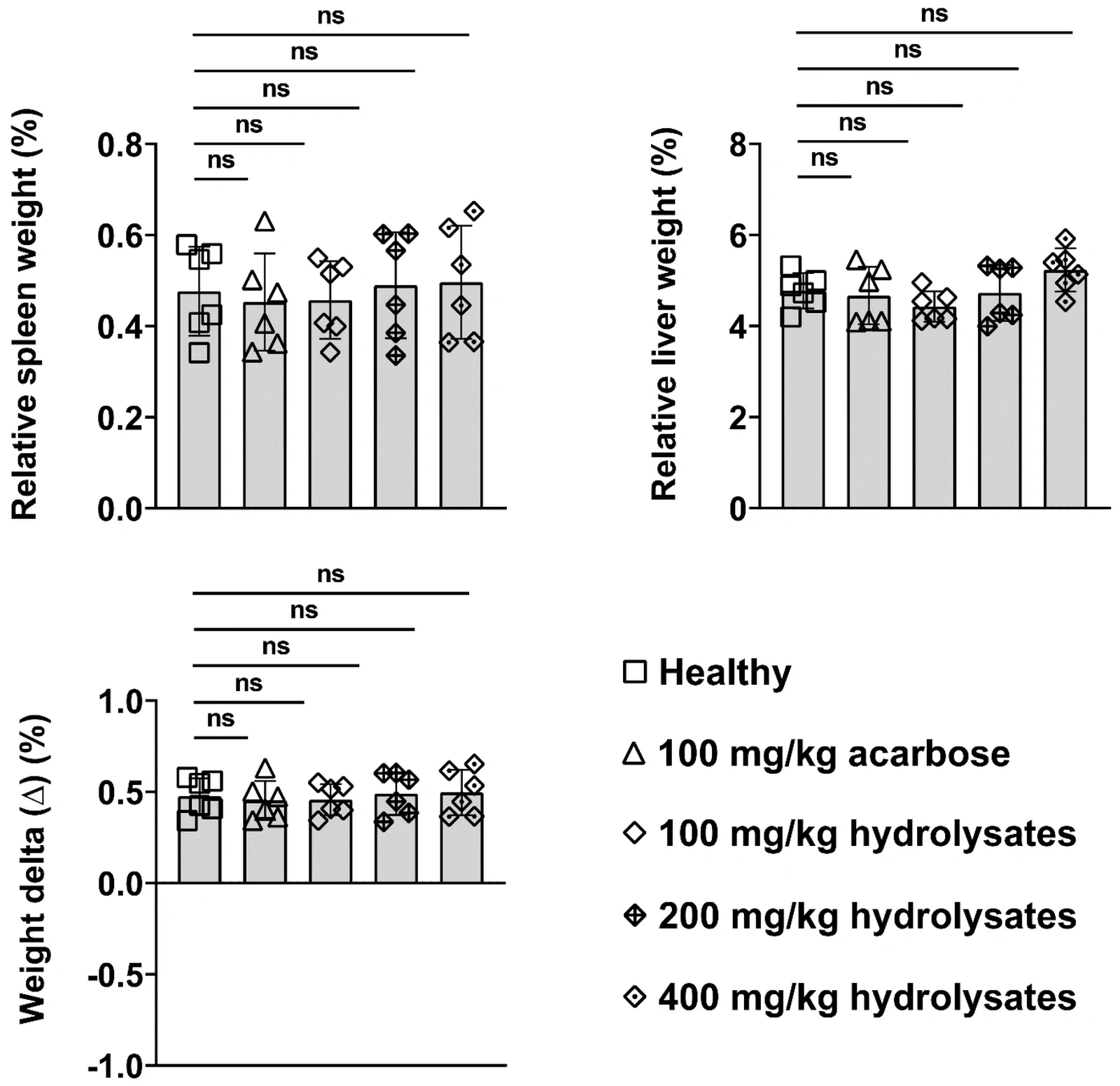
Changes in body weight, relative spleen weight, and relative liver weight in the subacute toxicity study. Data are presented as mean ± SEM (*n* = 6). Statistical analysis was performed using one-way ANOVA followed by Dunnett’s post hoc test. ns—not significant (*p* > 0.05).

**Figure 4:**
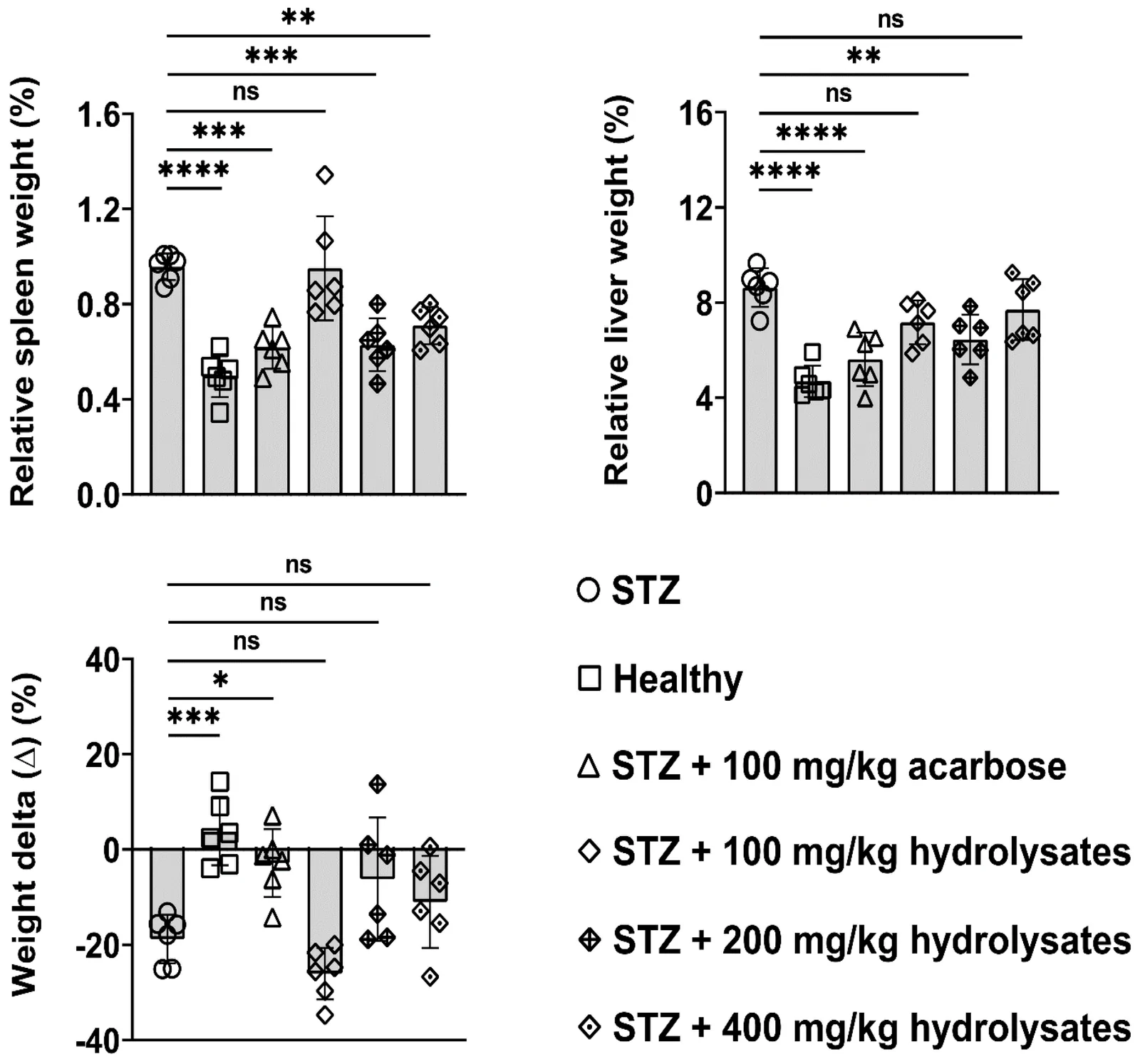
Effects of hemp seed cake hydrolysates on changes in body weight and relative spleen and liver weights in mice. Data are presented as mean ± SEM (*n* = 6; ns—not significant, * *p* < 0.05, ** *p* < 0.01, *** *p* < 0.001, **** *p* < 0.0001, compared with the streptozotocin (STZ) group). Statistical analysis was performed using one-way ANOVA followed by Dunnett’s post hoc test.

**Figure 5:**
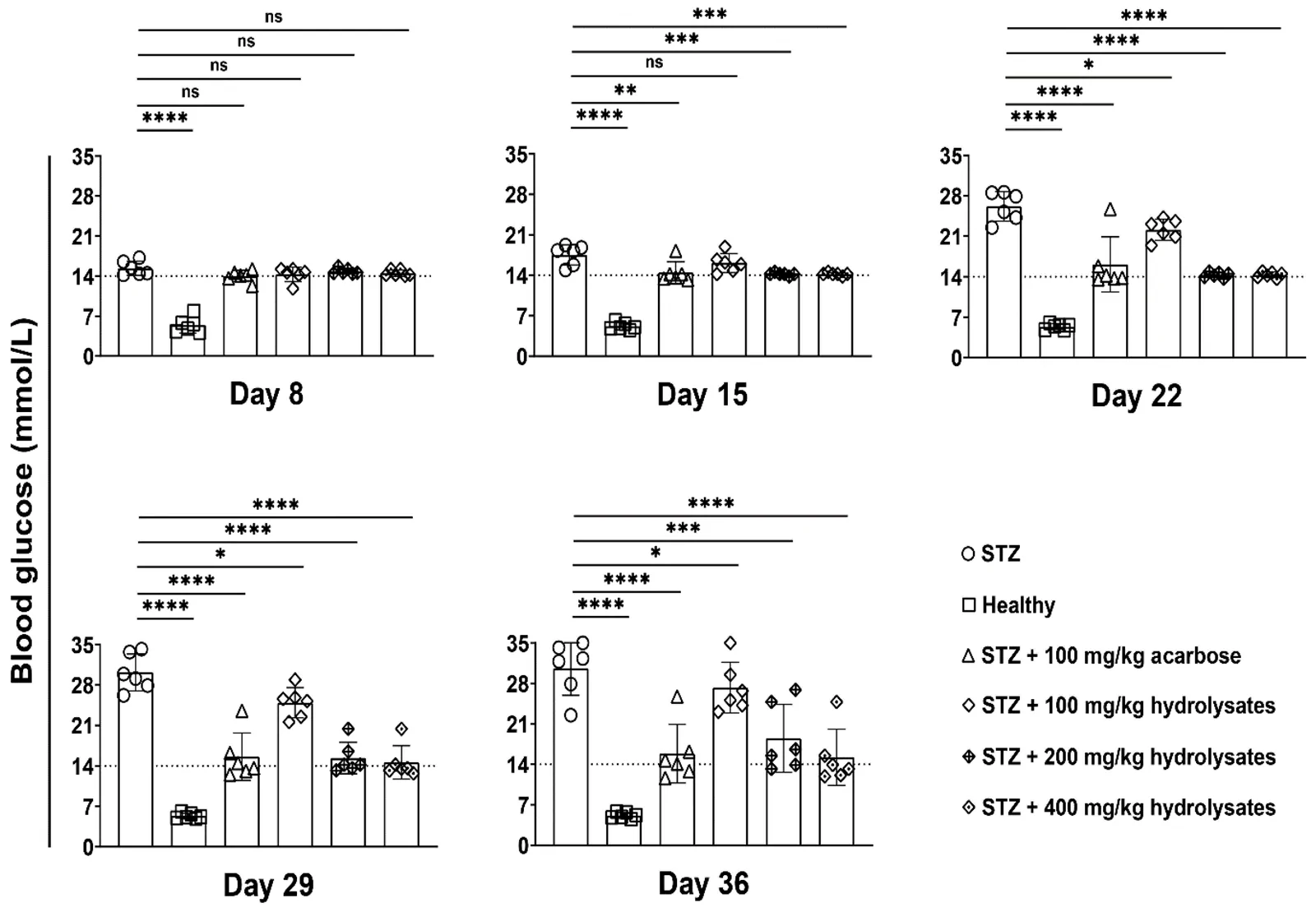
Effects of hemp seed cake hydrolysates on blood glucose levels in mice. Data are presented as mean ± SEM (*n* = 6; ns—not significant, * *p* < 0.05, ** *p* < 0.01, *** *p* < 0.001, **** *p* < 0.0001, compared with the streptozotocin (STZ) group). Statistical analysis was performed using one-way ANOVA followed by Dunnett’s post hoc test.

**Figure 6:**
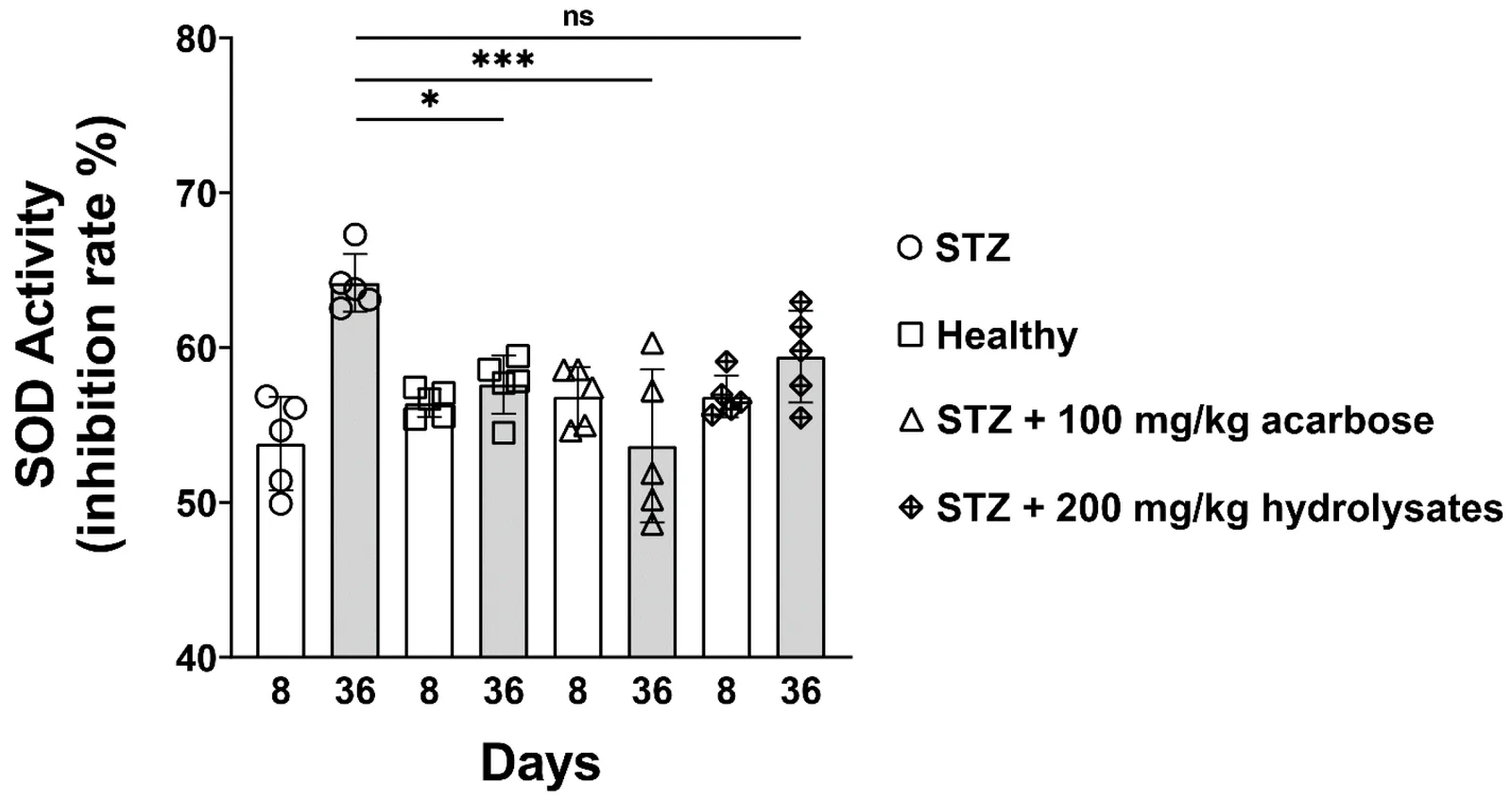
Effects of hemp seed cake hydrolysates on superoxide dismutase (SOD) activity. Data are presented as mean ± SEM (ns—not significant; * *p* < 0.05, *** *p* < 0.001, compared with the streptozotocin (STZ) group). Statistical analysis was performed using one-way ANOVA followed by Dunnett’s post hoc test.

**Figure 7:**
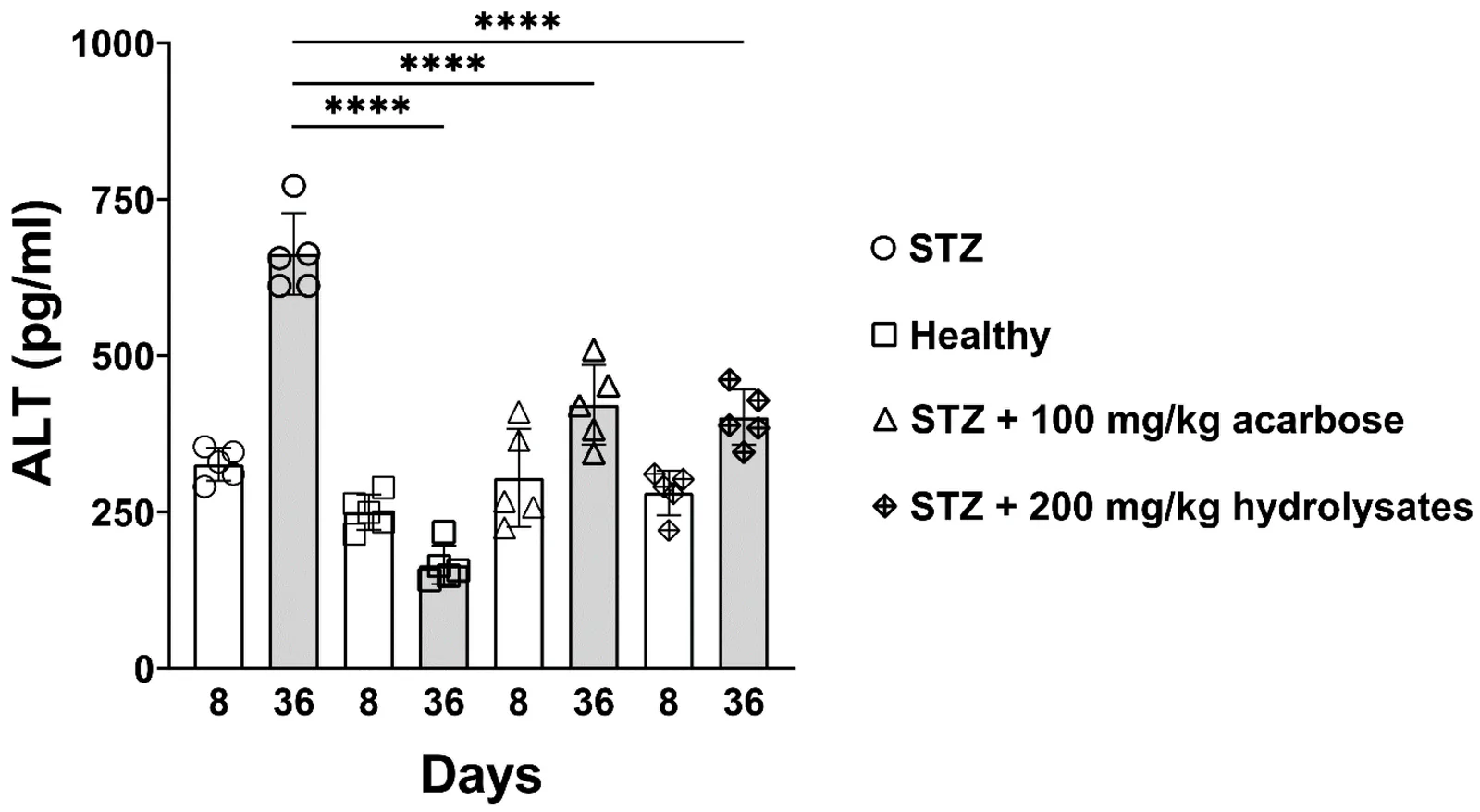
Effects of hemp seed cake hydrolysates on alanine aminotransferase (ALT) activity. Data are presented as mean ± SEM (**** *p* < 0.0001, compared with the streptozotocin (STZ) group). Statistical analysis was performed using one-way ANOVA followed by Dunnett’s post hoc test.

**Figure 8:**
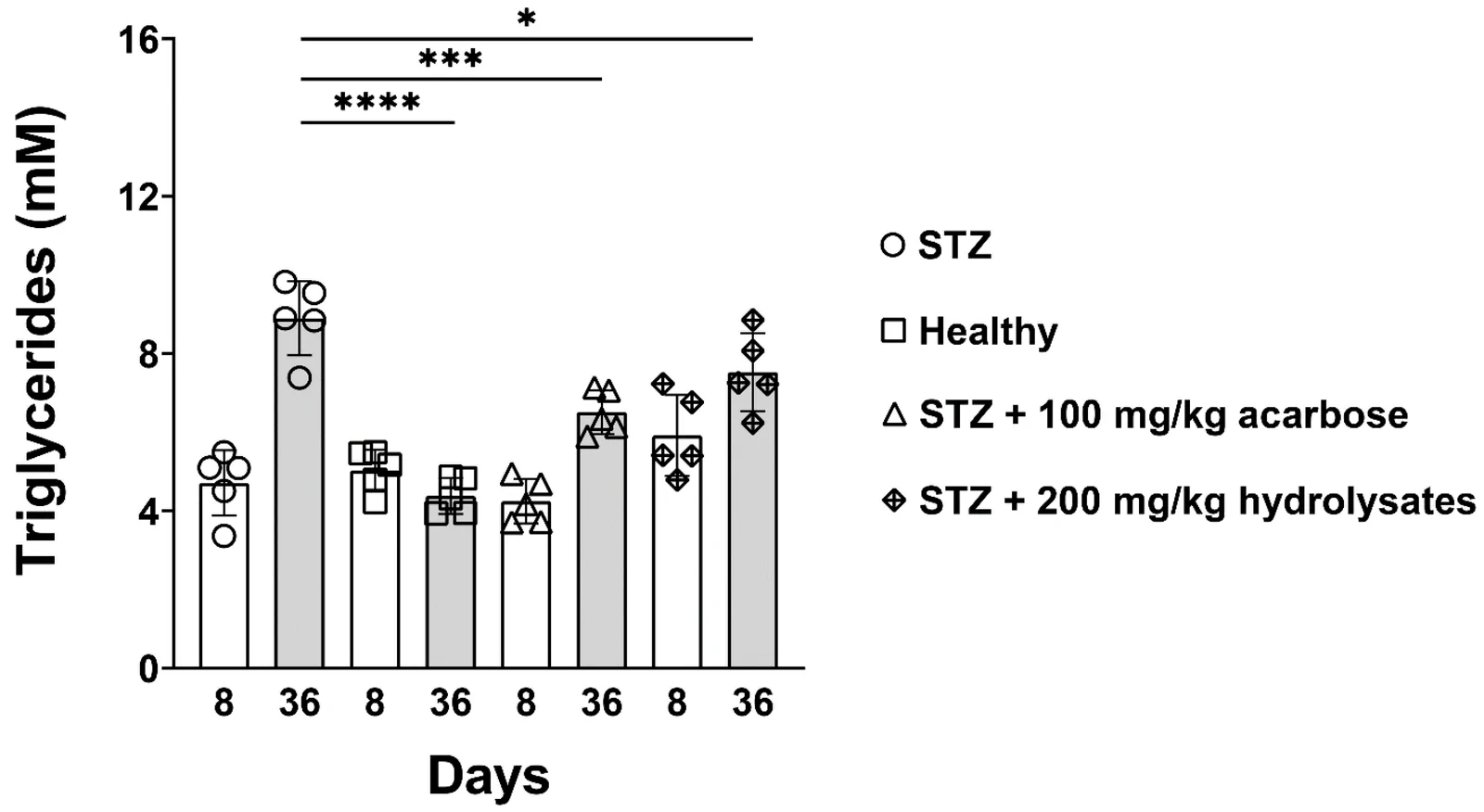
Effects of hemp seed cake hydrolysates on triglycerides. Data are presented as mean ± SEM (* *p* < 0.05, *** *p* < 0.001, **** *p* < 0.0001, compared with the streptozotocin (STZ) group). Statistical analysis was performed using one-way ANOVA followed by Dunnett’s post hoc test.

**Figure 9:**
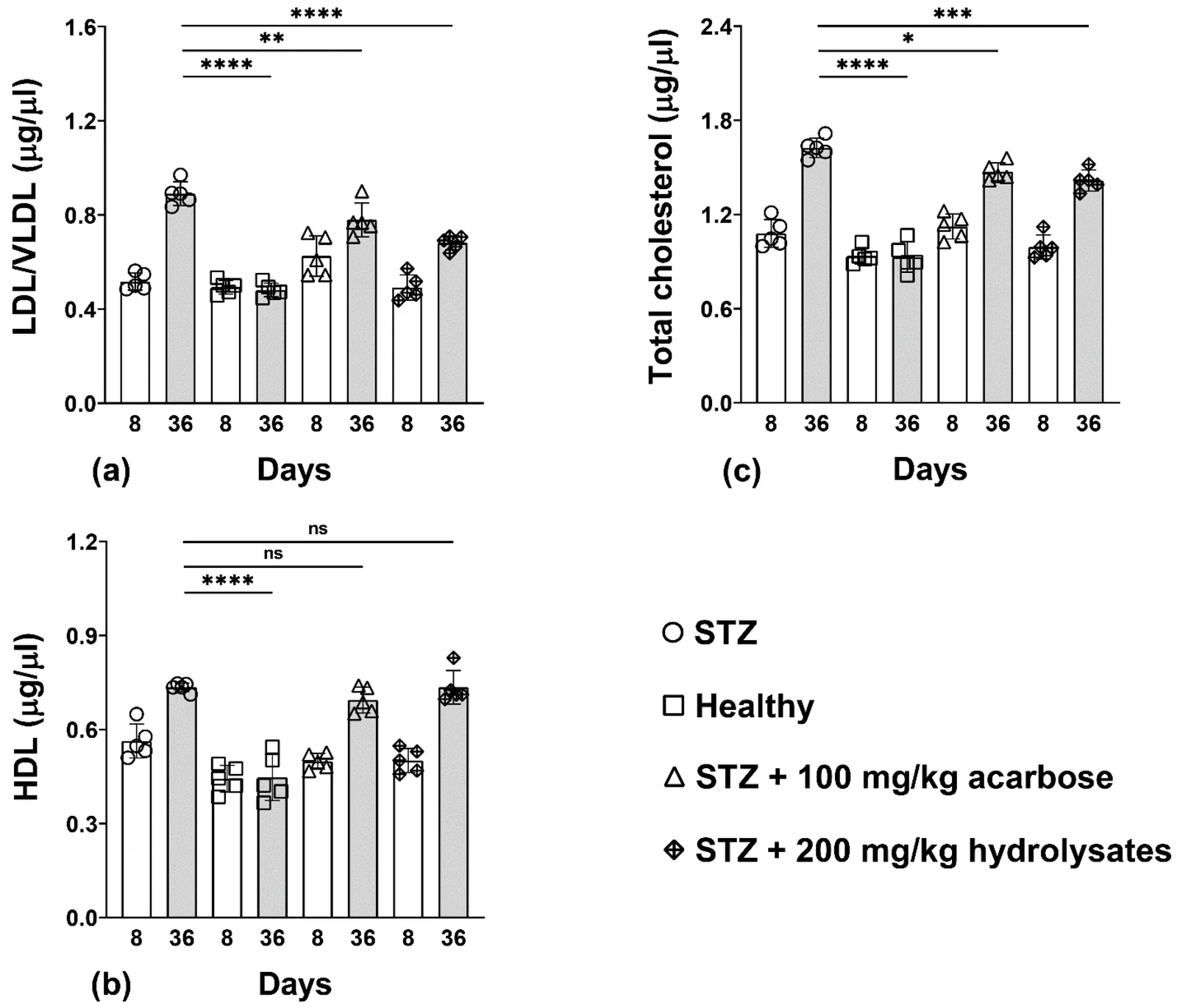
Effects of hemp seed cake hydrolysates on (**a**) low-density lipoprotein/very low-density lipoprotein (LDL/VLDL), (**b**) high-density lipoprotein (HDL), and (**c**) total cholesterol. Data are presented as mean ± SEM (ns—not significant, * *p* < 0.05, ** *p* < 0.01, *** *p* < 0.001, **** *p* < 0.0001, compared with the streptozotocin (STZ) group). Statistical analysis was performed using one-way ANOVA followed by Dunnett’s post hoc test.

## Data Availability

The original contributions presented in this study are included in the article. Further inquiries can be directed to the corresponding author.
